# Breastfeeding motivation in Pomerania: Survey of neonates in Pomerania (SNiP-Study)

**DOI:** 10.1186/s13006-016-0093-6

**Published:** 2017-01-06

**Authors:** Anja Lange, Anke Nautsch, Kerstin Weitmann, Till Ittermann, Matthias Heckmann

**Affiliations:** 1 Department of Pediatrics and Neonatology & Paediatric Intensive Care, University Medicine Greifswald, F.-Sauerbruchstr, 17475 Greifswald, Germany; 2 Institute of Community Medicine, Division of Health Care Epidemiology and Community Health, SHIP-KEF, University of Greifswald, Greifswald, Germany

**Keywords:** Breastfeeding duration; public health, Breastfeeding monitoring, Breastfeeding motivation, Breastfeeding promotion; breastfeeding initialisation

## Abstract

**Background:**

The Nationale Stillkommission was founded in Germany in 1994 to increase the acceptance of breastfeeding as the primary means of infant nutrition. Scientific studies like “Stillen und Säuglingsernährung (SuSe-Studie)”, and regional studies in Bavaria, Freiburg, Hamburg, and Berlin demonstrated breastfeeding initiation rates of 90 to 95%, but the total breastfeeding rate decreased to 25–61% after infants were 6 months old. One predictor of breastfeeding duration may be maternal motivation. The present study aimed to describe breastfeeding motivation.

**Methods:**

We analysed data collected in 2004–2008, during a previous study, the Survey of Neonates in Pomerania (SNiP). We retrieved data regarding maternal breastfeeding motivation, family environment, and socioeconomic factors. We constructed a quantitative breastfeeding-motivation score to identify factors involved in maternal breastfeeding.

**Results:**

Ninety five percent of mothers who gave birth in the study period and area provided information in the survey. The breastfeeding initiation rate was 88.4%. Mothers’ intentions to provide exclusive breastfeeding (only breast milk, no other liquids or infant formula) increased linearly from 71.9% in 2005 to 76.8% in 2008. Women motivated to provide exclusive breastfeeding were, on average, older, primiparous, and able to deliver spontaneously more often than women with less breastfeeding motivation. Furthermore, women with no motivation to provide exclusive breastfeeding and women that intended to provide breastfeeding combined with a complementary nutrition source had visited prenatal classes less frequently, had lower levels education, had lower average incomes, had a German nationality more often, and used tobacco more often than women motivated to provide exclusive breastfeeding.

**Conclusions:**

Breastfeeding intentions increased during the SNiP Study. This study identified several factors that might serve for targeted breastfeeding promotion in mothers younger than 25 years, mothers with low education, and multiparous mothers or women who have received a caesarean section. Furthermore, breastfeeding motivation might be enhanced during pregnancy and/or after delivery by providing prenatal classes.

## Background

Young and inexperienced mothers are often manipulated and confused by different views and recommendations about breastfeeding duration and initiation, the selection of an infant formula when suggested by different German or European paediatricians, and by industry, which promotes bottle-feeding and by the early introduction of complementary foods [1]. Nutrition is an important maternal issue, and mothers must decide between breastfeeding and bottle feeding. On one hand, most women have learned that breast milk is the optimal nutrition for their babies [1, 2]; and on the other hand, women like approaches that facilitate returning to work or regaining the quality of life they experienced before pregnancy. Sometimes, uncertainties or fears about a new situation, and breastfeeding in particular, play a role in maternal decision-making. Women are often sceptical about breastfeeding, suspecting that their babies will not be fed adequately [3, 4]. To increase the acceptance of breastfeeding as a normal means of baby nutrition, the National Breast Feeding Commission (Nationale Stillkommission) [5] was founded in Germany in 1994, was based on the WHO Innocenti Declaration, and published in 1990 [6]. In this context, recommendations about infant nutrition and the nutrition of breastfeeding mothers in Germany were prepared by the Nationale Stillkommission. The current recommendations regarding breastfeeding of newborns are as follows: (1) The first attempt to breastfeed should be performed during the first two hours after delivery; (2) for the first four to six months, the infant should be exclusively breastfed, and (3) complementary food should be introduced at five to seven months, and breastfeeding should continue until the first birthday or later [1]. When diabetes mellitus type I or allergic diseases, such as celiac diseases, are present, an introduction of complementary food may be recommended, like a special diet [1, 7]. These different statements, intensive industrial promotion of bottle-feeding, and the early introduction of complementary food, confuse young and inexperienced mothers [1]. In Germany, the breastfeeding initiation rates are 97 – 81.5% [4, 8, 9, 10]. During the first study in Germany, known as “Stillen und Säuglingsernährung” (SuSe-Study), conducted from 1997 to 1998, 91% of mothers initiated breastfeeding, but only 25% continued to breastfeed after four months [9]. Other studies showed similar results for breastfeeding iniation rates, including the national survey of children and youth (KiGGS) of 81.5% [8], the regional Bavarian study, “Stella” at 90% [10], and the Hamburger studies, Quasti 1 through 4 at 97% [4].

Beside data generation on breastfeeding rates at different time points, breastfeeding intention was also measured before and after delivery by standardized questionnaires that differed relevantly between studies. Breastfeeding intention was identified as a predictor of breastfeeding duration (r = 0.4) [11–14]. Furthermore, a lack of breastfeeding intention was important as a major risk factor for refusing breastfeeding initiation [9]. Some studies reported that mothers expressed a desire to breastfeed before or during pregnancy [4, 13, 15]. During the Stella study, mothers tended to initiate breastfeeding only after receiving information (OR 0.37; CI 0.23, 0.60) [10]. In other German studies, 92% (FreiStill) or 96% (Quasti) of mothers decided to breastfeed, either during or before pregnancy, and only 2 or 3% of women were indecisive [3, 4]. The high decison rate to breastfeed may be explained by the exclusion criteria. Those studies did not include underage mothers, mothers whose children had a birthweight < 2500 g, mothers who were transferred to a children’s hospital, mothers who had multiple births or delivered before 37 weeks gestation, or mothers that were not reachable for follow-up surveys [3, 4, 10].

Therefore, the aim of the present study was to describe breastfeeding motivation based on cross-sectional data collected in the Survey of Neonates in Pomerania (SNiP) [16]. The population based approach of this birth cohort allowed inclusion of all mothers and their babies with a extremely low selection bias. We investigated the intention to initiate breastfeeding, and the planned duration of breastfeeding. We also analysed perinatal and sociodemographic factors that might influence breastfeeding and to identify factors that might be modified to improve breastfeeding initialisation and motivation.

## Methods

### Study design

The present study was based on data from the population based birth cohort study, entitled the “Survey of Neonates in Pomerania (SNiP)”, which was conducted from 2002 to 2008. Details of the SNiP study were reported previously by Ebner et al. 2009 [16].

### Participants section

According to census data, 7220 babies were born in the study region of Pomerania in northeast Germany in the years 2002–2008. In the SNiP study, data were assessed from *n* = 6828 (95%) babies and their respective mothers (*n* = 6747), which yielded a high population coverage. Exclusions and non responders comprised *n* = 1556, of 6828 babies. A minimum dataset was compiled that contained data on the health status of these newborns and their mothers, but lacked detailed information on environmental parameters.

A non responder analysis yielded statistically significant results between participants and non participants in several variables. Participants were significantly older, less frequently single and more frequently primiparae. The participants delivered singletons less frequently and their newborn’s gestational age was significantly shorter. There was no statistically significant difference of participation due to the mode of birth or the frequency of newborns that were delivered to a special unit. However, the effect sizes of the statistically significant differences were all under the threshold of 0.1, indicating that the effect was not meaningful [16].

### Procedures

Comprehensive data on newborn infants and their mothers, regarding neonatal health, morbidity, and mortality, was collected to calculate the prevalence of major neonatal diseases, risk factors, and confounding conditions like socioeconomic background, on both cross-sectional and prospective bases. Physicians especially trained for the study collected the data at the three participating hospitals. Questionnaires were filled in at a single time point 2–4 days after delivery. We collected personal data, medical records (149 variables) and data from a personal interview (84 variables).

Mothers were also asked to complete a questionnaire during their stay in the ward and return it to the medical staff before discharge. This questionnaire included questions about the parents’ social background, lifestyle, any preventive examinations and all questions about breastfeeding (40 variables). Questionnaires were filled in by the mothers at a single time point 2–4 days after delivery.

Breastfeeding was defined as follows:

Exclusive breastfeeding: only breast milk, no other liqueds or infant formula. Partial breastfeeding: breast milk plus other feeds such as liquids, infant formula.

Questions about breastfeeding:

There were open and closed questions were referring to breastfeeding.

### Breastfeeding motivation score (BMFS)

The breastfeeding motivation score (BFMS) was calculated with the following variables:

- Intended breastfeeding during the first six months: exclusive, defined as breast milk provides the only source of nutrition = 5 points; partial, defined as breast milk combined with alternative sources of nutrition = 2 points; none, defined as no breast milk provided = 0 points.
- Intended duration of breastfeeding: more than 6 months = 5 points; 5–6 months = 3 points; 1–4 months = 2 points; no information given = 0 points.
- Mothers were asked to give reasons for or against breastfeeding their newborns. For the calculation of the score 1 point was given to each mother who gave at least one reason for breastfeeding her child. Mothers who did not respond to this question were given 0 points and mothers who stated reasons against breastfeeding (e.g. mother is smoking, lack of time) were given -1 points.

The BFMS was calculated as the sum of a + b + c + 1, and it covered a range of 0 – 11 points.

Low alcohol consumption was indicated by a value ≤ 3, and suspected consumption was indicated with a value > 3. Tobacco consumption classifications were: low for values ≤ 4 and excessive for values > 5.

### Ethics and data protection

All mothers provided written informed consent to participate in the study. Participation was on a voluntary basis and without payment. The study was approved by the Ethics Committee of the Ernst Moritz Arndt University, Greifswald, as well as the data safety commissioner of the federal state of Mecklenburg-Western Pomerania. The collected data were anonymised and stored in an Access database and managed by a independent university trust agency.

### Statistical methods

We stratified data by the proposed motivation to perform breastfeeding. Continuous data are reported as the median with 25th and 75th percentiles; categorical data are expressed as the absolute number and percentage. Bivariate associations of potential risk factors with breastfeeding motivation and duration were calculated with multinomial logistic regression. Bivariate associations of potential risk factors with the BFMS were calculated with Poisson regression. The relative risk (RR) derived from the Poisson regression model explained the relative risk for a one point increase in the BFMS. All the bivariate considered variables were associated with the BFMS in a multivariable Poisson regression model. A backward elimination procedure was applied to the Poisson regression model to ensure that only relevant variables were retained in the model. In all analyses, *p* < 0.05 was considered statistically significant. All analyses were carried out with Stata 14.1 (Stata Corporation, College Station, TX, USA).

## Results

The present study included data from 4047 mothers of neonates delivered between January 2004 and November 2008. Out of the mothers included in SNiP, information on breastfeeding intention was available from 3586 (88.7%) mothers, which formed the basis for the present analysis.

Two thoursand, six hundred and eight women intended to exclusively breastfeed; 553 women to partially breastfeed with other food, and 407 women with no breastfeeding at all (Table 1).

**Table 1:** Characteristics of the study population, stratified by proposed breastfeeding plan during the first six months after birth

Characteristics	Proposed breastfeeding plan		
Breastfeeding after delivery	exclusive (*n* = 2608)	partial (*n* = 553)	none (*n* = 407)
Proposed duration of breastfeeding			
no information	1645 (62.8%)	365 (65.9%)	407 (100.0%)
≤4 months	164 (6.3%)	133 (24.0%)	0 (0.0%)
5 – 6 months	647 (24.7%)	50 (9.0%)	0 (0.0%)
>6 months	162 (6.2%)	6 (1.1%)	0 (0.0%)
Breastfeeding motivation score	7 (7; 10)	4 (4; 6)	1 (1; 2)
Age of mother > 25 years	1710 (65.6%)	304 (55.2%)	223 (54.8%)
Multiparous	1116 (42.8%)	223 (40.5%)	260 (64.2%)
Birth mode	1826 (70.0%)	349 (63.1%)	250 (61.7%)
spontaneous	678 (26.0%)	180 (32.6%)	150 (37.0%)
caesarean section other	104 (4.0%)	24 (4.3%)	5 (1.2%)
Year of delivery			
2004 – 2005	1067 (40.9%)	248 (44.9%)	178 (43.8%)
2006 – 2008	1544 (59.1%)	305 (55.2%)	229 (56.3%)
Prenatal class	1384 (54.4%)	190 (35.1%)	95 (24.0%)
German nationality	2538 (97.2%)	541 (97.8%)	403 (99.3%)
Education level			
low	298 (11.7%)	96 (17.9%)	128 (33.2%)
medium	1376 (53.8%)	340 (63.3%)	235 (60.9%)
high	882 (34.5%)	101 (18.8%)	23 (6.0%)
Average income; €/month	866 (505; 1342)	671 (438; 1163)	518 (359; 866)
Married	1041 (39.9%)	173 (31.2%)	131 (32.4%)
Gestational week before week 37	306 (11.7%)	80 (14.5%)	48 (11.8%)
Alcohol consumption			
once a month	613 (23.6%)	122 (22.4%)	89 (22.1%)
more than once a month	77 (3.0%)	8 (1.5%)	4 (1.0%)
Smoking during pregnancy	321 (12.7%)	128 (24.3%)	165 (42.9%)
Inpatient stay during pregnancy	832 (31.9%)	209 (37.8%)	135 (33.3%)

Continuous data are expressed as the median (25th; 75th percentiles); categorical data are expressed as absolute numbers and percentages

### Motivation for exclusive breastfeeding: univariate analysis

The motivation for exclusive breastfeeding was positively associated with the age of the mother, attending prenatal classes, medium or high education level, average income, being in a solid relationship, and alcohol intake more than once a month. Conversely, the motivation for exclusive breastfeeding was inversely associated with being multiparous, delivery by caesarean section, German nationality, and smoking during pregnancy (Table 2). In comparison, mothers motivated to perform partial breastfeeding were significantly less often multiparous, attended prenatal classes significantly more often, had significantly higher education, had a significantly higher equivalent income, and were significantly less often smokers than mothers with no motivation to breastfeed. The proposed duration of breastfeeding was significantly associated with age of the mother, parity, birth mode, year of delivery, attending prenatal classes, German nationality, education level, average income, partnership, and alcohol consumption. The BFMS was positively associated with the age of the mother, delivery between 2006 and 2008, attending prenatal classes, a medium or high education level, a high average income, being in a permanent relationship, and alcohol intake more than once a month. The BFM score was inversely associated with being multiparous, delivery by caesarean section, German nationality, and smoking during pregnancy. Mothers delivering before week 37 had no significantly different breastfeeding motivation than mothers who delivering after week 37 (Table 2).

**Table 2:** Association between potential risk factors and breastfeeding motivation

Variable	Proposed breastfeeding plan RR (95% CI)		Proposed duration of breastfeeding RR (95% CI)			BFMS RR (95% CI)
	exclusive vs. none	partial vs. none	1-4 months vs. no info	5-6 months vs. no info	>6 months vs. no info	
Age of mother > 25 years	1.57 (1.27; 1.94)*	1.02 (0.79; 1.31)	0.66 (0.52; 0.84)*	1.08 (0.91; 1.29)	1.09 (0.78; 1.51)	1.07 (1.04; 1.09)*
Multiparous	0.42 (0.34; 0.52)*	0.38 (0.29; 0.49)*	0.72 (0.56; 0.92)*	0.59 (0.50; 0.70)*	1.16 (0.84; 1.59)	0.91 (1.04; 1.09)*
Birth mode						
caesarean vs. spont.	0.62 (0.50; 0.77)*	0.86 (0.66; 1.13)	1.14 (0.87; 1.48)	1.07 (0.88; 1.29)	1.09 (0.78; 1.54)	0.96 (0.93; 0.99)*
other vs. spont.	2.85 (1.15; 7.05)*	3.44 (1.29; 9.13)*	1.27 (0.68; 2.37)	1.67 (1.12; 2.50)*	0.73 (0.26; 2.04)	1.07 (1.01; 1.14)*
Year of delivery						
2006/08 vs. 2004/05	1.12 (0.91; 1.39)	0.96 (0.74; 1.24)	1.00 (0.79; 1.28)	1.12 (0.94; 1.32)	1.44 (1.04; 2.00)*	1.04 (1.01; 1.07)*
Prenatal class	3.78 (2.97; 4.83)*	1.72 (1.28; 2.29)*	0.92 (0.72; 1.18)	1.98 (1.66; 2.36)*	1.28 (0.93; 1.75)	1.21 (1.18; 1.24)*
German vs. foreign nationality	0.26 (0.08; 0.84)*	0.34 (0.09; 1.20)	3.72 (0.90; 15.29)	1.32 (0.72; 2.42)	0.30 (0.16; 0.55)*	0.90 (0.83; 0.97)*
Education level						
medium vs. low	2.52 (1.96; 3.23)*	1.92 (1.41; 2.64)*	1.61 (1.12; 2.30)*	2.21 (1.65; 2.98)*	1.05 (0.64; 1.71)	1.21 (1.16; 1.26)*
high vs. low	16.5 (10.4; 26.2)*	5.86 (3.47; 9.89)*	0.92 (0.60; 1.40)	2.36 (1.72; 3.23)*	2.02 (1.23; 3.30)*	1.39 (1.34; 1.45)*
Average income;	1.15 (1.12; 1.19)*	1.09 (1.06; 1.13)*	0.97 (0.95; 0.99)*	1.02 (1.01; 1.04)*	1.03 (0.99; 1.06)*	1.01 (1.00; 1.02)*
€/per month						
Solid partnership	1.39 (1.11; 1.73)*	0.95 (0.72; 1.25)	0.68 (0.52; 0.88)*	0.83 (0.69; 0.99)*	0.93 (0.68; 1.29)	1.03 (1.01; 1.05)*
Gestational week	1.01 (0.73; 1.39)	0.79 (0.54; 1.16)	1.30 (0.87; 1.93)	1.10 (0.85; 1.43)	1.28 (0.77; 2.15)	1.03 (0.99; 1.07)
>37 vs. ≤ 37						
Lung maturation	1.26 (0.84; 1.89)	1.55 (0.97; 2.48)	0.91 (0.58; 1.42)	1.16 (0.86; 1.54)	0.91 (0.51; 1.64)	1.02 (0.98; 1.07)
Alcohol consumption						
once a month vs. none	1.12 (0.87; 1.44)	1.03 (0.75; 1.40)	1.20 (0.91; 1.58)	1.34 (1.10; 1.63)*	1.03 (0.70; 1.51)	1.04 (1.01; 1.07)*
> once a month	3.13 (1.14; 8.62)*	1.50 (0.45; 5.02)	0.32 (0.08; 1.34)	1.85 (1.13; 3.01)*	3.22 (1.63; 6.35)*	1.19 (1.10; 1.28)*
Smoking during pregnancy	0.19 (0.15; 0.24)*	0.43 (0.32; 0.57)*	-^a^	-^a^	-^a^	0.63 (0.60; 0.65)*
Inpatient stay during pregnancy	0.93 (0.75; 1.17)	1.22 (0.93; 1.59)	1.02 (0.79; 1.32)	0.99 (0.82; 1.18)	0.92 (0.65; 1.29)	0.98 (0.96; 1.01)

Data was analysed with a bivariate multinomial logistic regression model for the outcomes of proposed breastfeeding plan and proposed duration of breastfeeding and with a bivariate Poisson regression model for the breastfeeding motivation score (BFMS). *RR* = risk ratio, *CI* = confidence interval; *p < 0.05

^a^None of the women that smoked provided information on the proposed duration of breastfeeding

### Motivation of breastfeeding: multiple logistic regression analysi**s**

The multivariable Poisson regression with backward elimination (using all variables described in Table 1 as independent variables and the BFMS as outcome) showed that the following variables remained statistically significant: being multiparous (RR 0.94; 95% confidence interval [CI] 0.92, 0.97; *p* ≤ 0.001), caesarean section vs. spontaneous delivery (RR = 0.95; 95% CI 0.92, 0.98; *p* = 0.001), smoking during pregnancy (RR 0.68; 95% CI 0.65, 0.71; *p* < 0.001), delivery in 2006 – 2008 vs. 2004 – 2005 (RR 1.04; 95% CI 1.01, 1.06; *p* = 0.010), attending prenatal classes (RR 1.08; 95% CI 1.05, 1.11; *p* < 0.001), German nationality (RR 0.92; 95% CI 0.84, 0.99; *p* = 0.036), medium vs. low education level (RR 1.05; 95% CI 1.01, 1.10; *p* = 0.030), high vs. low education level (RR 1.11; 95% CI 1.05, 1.17; *p* < 0.001), alcohol intake once a month (RR 1.04; 95% CI 1.01, 1.07; *p* = 0.014), and alcohol intake more than once a month (RR 1.15; 95% CI 1.06, 1.24; *p* = 0.001) (Table 3).

**Table 3:** **-**Risk factors for the breastfeeding motivation score

	Breastfeeding motivation score Relative risk (95% confidence interval)
Multipara v. primipara	0.94 (0.9, 0.97); *p* < 0.001
Sectio vs. spontaneous delivery	0.95 (0.92, 0.98); *p* = 0.001
Smoking during pregnancy	0.68 (0.65, 0.71); *p* < 0.001
Delivery in 2006 – 2008 vs. 2004 - 2005	1.04 (1.01, 1.06); *p* = 0.010
Attending prenatal classes	1.08 (1.05, 1.11); *p* < 0.001
German nationality	0.92 (0.84, 0.99); *p* = 0.036
Medium vs. low education level	1.05 (1.01, 1.10); *p* = 0.030
High vs. low education level	1.11 (1.05, 1.17); *p* < 0.001
Alcohol once a month vs. no alcohol	1.04 (1.01, 1.07); *p* = 0.014
Alcohol more than once a month vs. no alcohol	1.15 (1.06, 1.24); *p* = 0.001

Data was analyzed by multivariable Poisson regression initially including all potential risk factors for the breastfeeding motivation score. In the table only those variables are included which were kept in the model after a backward elimination procedure

## Discussion

In the present study, we investigated factors that potentially influenced the motivation for maternal breastfeeding at the time of delivery, in a large sample of 3586 mothers. Based on the inclusion criteria, women were admitted to the regional birth collective study over a period of 47 months [16]. The women expressed a plan, based on their motivation, for breastfeeding during the first six months. By answering both open and closed questions, women thought about their attitudes towards the natural nutrition of babies through breastfeeding. The breastfeeding motivation score was 88.4% for the whole breastfeeding period compared to 90–97% in the studies in Bavaria, Berlin, Hamburg, and Freiburg, or the SuSe-Studie [3, 4, 6, 10]. Incontrast to these studies, this may be explained by our approach to recruite preferably all mothers including those with low birth weight or preterm newborns, mothers who were transferred to a children’s hospital, mothers who had multiple births, or mothers that were not reachable for follow-up surveys.

The SNiP study found motivation to initialise breastfeeding among women who were primiparous, > 25 years of age, educated at high levels, and willing to participate in prenatal classes, all consistent with previous studies on this topic [3, 12]. In accordance with Rasenack [3], Deneke et al., and Kohlhuber et al. [4, 10], we demonstrated that mothers who underwent caesarean sections had lower motivation for breastfeeding than mothers who delivered spontaneously or by other delivery methods.

In contrast to Kohlhuber [10] being preterm was not a risk factor for not breastfeeding. (Table 3). In contrast to previous studies we did not detect an association between gestational week of delivery and breastfeeding motivation (Table 2, Table 3).

We confirmed the factors previously described in the SuSe-Studie [9] that were associated with a short breastfeeding duration (only four months), such as a lower education level or maternal age < 25 years. The motivation for exclusive breastfeeding was positively associated with the age of the mother, attending prenatal classes, medium or high level of education, equivalent income, being in a solid relationship, and alcohol intake more than once a month. Conversely, the motivation for exclusive breastfeeding was inversely associated with being multiparous, delivery by caesarean section, German nationality, and smoking during pregnancy (Table 2). The recommendations of the National Breastfeeding Commission for exclusive breastfeeding until five months after birth were not met by women in the SNiP study that intended to breastfeed up to four months after birth; however, the intention of those women were consistent with the Commision’s recommendation that every form of breastfeeding should be endorsed [1].

In this survey, women were not asked about the actual duration of breastfeeding. However several studies showed a positive association between positive breastfeeding motivation during pregnancy and actual breastfeeding duration [3, 13–15].

### Limitations and strengths

The main limitation of the study is that we did not determine the duration of breastfeeding. However, it was shown in several studies as outlined before that breastfeeding motivation correlated to the breastfeeding rate [13]. Furthermore, this study may include a selection bias, due to the choice of the study region and the method of recruitment (voluntary participation). Voluntary participation may have favoured the inclusion of participants with predominantly positive responses or with responses that reflected socially expected views. This impact was limited by the use of an anonymised questionnaire [16].

The strength of the study is the inclusion of a large number of women and it’s population-based character which allows generalisation of results to a population.

## Conclusion

The SNiP-Study is a population based studyt to provide information on breastfeeding motivation. In our study, breastfeeding motivation was lower than in other reports from Germany because of our population-based approach. Our findings suggested that breastfeeding motivation might be enhanced during pregnancy and/or after delivery by providing prenatal classes and informing mothers younger than 25 years, mothers with low level of education, and multiparous mothers on the benefits of breastfeeding. Women who have a caesarean section are a special target for postpartum counselling. Care providers should focus their efforts to encourage primiparous mothers and mothers who intend to partially breastfeed methods to increase breastfeeding durations to six months after birth. The utilisation of our breastfeeding motivation score provided an overview of breastfeeding motivation among women in a large population.

## Abbreviations

AUDIT-C: Alcohol use disorder identification test
BzgA: Bundeszentrale für gesundheitliche aufklärung (Federal centre for health education)
FTND: Fagerström test of nicotine dependence
KiGGS: Kinder- und Jugendgesundheitssurvey (German health interview and examination survey for children and adolescents)
OR: Odds ratio
*P*: Probability
Pp: Postpartum
R: Correlation
SNiP: Survey of neonates in Pomerania
SuSe-Studie: “Stillen und säuglingsernährung” study
WHO: World health organisation
